# Genome Sequence of *Stachybotrys chartarum* Strain 51-11

**DOI:** 10.1128/genomeA.01114-15

**Published:** 2015-10-01

**Authors:** Doris A. Betancourt, Timothy R. Dean, Jean Kim, Josh Levy

**Affiliations:** a United States Environmental Protection Agency, National Risk Management Research Laboratory, Air Pollution Prevention and Control Division, Durham, North Carolina, USA; b RTI International, Durham, North Carolina, USA

## Abstract

The *Stachybotrys chartarum* strain 51-11 genome was sequenced by shotgun sequencing utilizing Illumina HiSeq 2000 and PacBio technologies. Since *S. chartarum* has been implicated as having health impacts within water-damaged buildings, any information extracted from the genomic sequence data relating to toxins or the metabolism of the fungus might be useful.

## GENOME ANNOUNCEMENT

*Stachybotrys chartarum* is a saprophytic fungus with worldwide distribution. In nature, it is isolated from soil, seeds, and decaying organic matter. Indoors, *S. chartarum* grows on wet cellulose-containing building material, such as gypsum wallboard, cardboard, and ceiling tiles (1).

*S. chartarum* is among the indoor fungal population frequently identified in water-damaged buildings, and it has been linked to damp building-related illnesses (DBRIs) (2). This fungus is capable of producing mycotoxins, of which the macrocyclic trichothecenes are among the most toxic (3, 4).

*S. chartarum* toxigenic strain 51-11, an environmental isolate obtained from the cluster of idiopathic pulmonary hemorrhage cases (Cleveland, OH) (5), is part of the RTI International (Research Triangle Park, NC) microbial collection assigned a unique identification number and stored at -80°C for long-term preservation. The fungal spores were grown in potato dextrose broth (PDB) (Becton, Dickinson & Company, Sparks, MD) and were shaken at 25°C. The resulting fungal mycelia were harvested using a sterile Miracloth (EMD Millipore, Darmstadt, Germany) and washed with sterile distilled water. The mycelia were flash-frozen in liquid nitrogen and stored at -20°C until the genomic DNA was isolated using a protocol described by Kohler et al. (6).

Two genomic DNA libraries were produced for Illumina sequencing: one paired-end library (insert size, 350 to 390 bp) and one mate-paired library (insert size, 100 to 5000 bp). These two libraries were sequenced on an Illumina HiSeq 2000 (Illumina, San Diego, CA) at Argonne National Laboratory (Lemont, IL). The machine produced 460× coverage, and approximately 100× of that coverage was actually used for the assembly, although the whole data set was exploited for error correction. The genome size was assessed using *k*-mer-counting tools, which pointed to a genome size of approximately 40 Mb. The resulting 40-Mb error-corrected reads were assembled on a large-memory machine using MIRA (http://genome.cshlp.org/content/14/6/1147.full). The resulting contigs were hand-edited for quality. SSPACE basic 2.0 (Leiden, The Netherlands) was run on the mate-pair data to try to close gaps and produce better scaffolds. The resulting assembly contained 2,843 scaffolds. Higher-molecular-weight DNA for PacBio long-read sequencing was also produced to close gaps in the Illumina assembly. Nine sequencing runs were performed, and after standard quality assurance/quality control cutoffs were applied, there were 284,625 reads, with ~736 million bases total (~2,600 bases per read), or ~18× coverage. Two hybrid assembly methods were compared, SSPACE-LongRead and PBJelly. Multiple combinations using both programs were performed on a supercomputing cluster, and the quality of assembly was assessed using standard metrics (*N*_50_ and *N*_90_). Three iterations of SSPACE-LongRead alone (not in combination with PBJelly) showed the highest-quality assembly, with the final number of scaffolds at 1,435, or approximately half of the Illumina assembly alone.

### Nucleotide sequence accession numbers.

This whole-genome shotgun project has been deposited at DDBJ/EMBL/GenBank under the accession no. LDEE00000000. The version described in this paper is version LDEE01000000.
